# Basic self-disturbances are associated with Sense of Coherence in patients with psychotic disorders

**DOI:** 10.1371/journal.pone.0230956

**Published:** 2020-04-15

**Authors:** Ingrid Hartveit Svendsen, Merete Glenne Øie, Paul Møller, Barnaby Nelson, Ingrid Melle, Elisabeth Haug

**Affiliations:** 1 Department of Acute Psychiatry and Psychosis Treatment, Division of Mental Health, Innlandet Hospital Trust, Reinsvoll, Norway; 2 Faculty of Medicine, University of Oslo, Blindern, Oslo, Norway; 3 Department of Psychology, University of Oslo, Oslo, Norway; 4 Division of Research, Innlandet Hospital Trust, Brumunddal, Norway; 5 Department of Mental Health Research and Development, Division of Mental Health and Addiction, Vestre Viken Hospital Trust, Drammen, Norway; 6 Orygen, The National Centre of Excellence in Youth Mental Health, The University of Melbourne, Victoria, Australia; 7 Centre for Youth Mental Health, The University of Melbourne, Victoria, Australia; 8 NORMENT KG Jebsen Centre for Psychosis Research, Institute of Clinical Medicine, University of Oslo, Oslo, Norway; 9 Division of Mental Health and Addiction, Oslo University Hospital, Oslo, Norway; Department of Psychiatry and Neuropsychology, Maastricht University Medical Center, NETHERLANDS

## Abstract

**Background:**

The *Sense of Coherence* (SOC) theory gives a possible explanation of how people can experience subjective good health despite severe illness. Basic self-disturbances (BSDs) are subtle non-psychotic disturbances that may destabilize the person’s sense of self, identity, corporeality, and the overall ‘grip’ of the world.

**Aim:**

Our objective was to investigate associations between BSDs and SOC in patients with psychotic disorders.

**Design:**

This is a cross-sectional study of 56 patients diagnosed with psychotic disorders inside and outside the schizophrenia spectrum (35 schizophrenia, 13 bipolar, and eight other psychoses). SOC was measured using Antonovsky’s 13-item SOC questionnaire, and BSDs were assessed using the Examination of Anomalous Self-Experience (EASE) manual. Diagnosis, symptoms, and social and occupational performance were assessed using standardized clinical instruments.

**Results:**

We found a statistically significant correlation (r = ) between high levels of BSDs and low levels of SOC (r = -0.64/*p*<0.001). This association was not influenced by diagnostics, clinical symptoms or level of functioning in follow-up multivariate analyses.

**Conclusion:**

A statistically significant association between BSDs and SOC indicates that the presence and level of self-disturbances may influence the person's ability to experience life as comprehensive, manageable and meaningful. However, the cross-sectional nature of the study precludes conclusions regarding the direction of this association.

## Introduction

The personal experience of health is complex and dynamic and relies on physical, social and mental factors [1]. According to the salutogenesis theory, an approach focusing on factors that support health and wellbeing, the experience of health is on a continuum from good health to poor health dependent on the individual’s internal and external coping resources [2]. Aron Antonovsky’s "Sense of Coherence (SOC)" theory is used to explain the apparent contradiction that people with severe illnesses report a subjective experience of good health.

According to this theory, the SOC comprises three theoretically separable but closely interwoven core components that include:

1) Comprehensibility: Refers to the extent to which a person perceives internal and external stimuli as rationally understandable and as information that is orderly, coherent, clear, structured rather than noise.

2) Manageability: Refers to the degree to which a person feels that there are resources at his/her disposal that can be used to meet the requirements of the stimuli that bombards him/her.

3) Meaningfulness: Refers to the extent to which a person feels that life has emotional meaning, that at least some of the problems faced in life are worth commitment and dedication, and are seen as challenges rather than as burdens [3].

These three core components can vary over time. A person can experience life as manageable but not explainable or meaningful, and later on as meaningful but not predictable and manageable [4]. A high SOC indicates the ability to make sense of the world, in a way that facilitates successful coping with the countless and complex stressors we confront throughout life [2].

Longitudinal studies of healthy teenagers [5], students [6]and unselected general population samples [7]show that a person's SOC is relatively stable throughout life. A 13-year follow-up study of 552 individuals followed from childhood to adulthood, found that SOC tended to increase with age and was most stable in those with a high SOC at study baseline [8]. While SOC is not seen as a personality trait, it is closely related to other personal resources such as self-esteem [9, 10]

Both quantitative and qualitative studies support the notion that a high SOC contributes to a high subjective quality of life [11]. Good health, and in particular, good mental health is also related to a high SOC. The original SOC theory takes the possibility of people refusing to acknowledge facts into account; i.e. the possibility of having a "false" SOC [3]. Loss of insight, especially the lack of awareness of positive- and cognitive symptoms, is common in patients with schizophrenia [12, 13]. Loss of insight could thus influence the association between SOC and mental health in schizophrenia. However, a study of 136 patients with schizophrenia or schizoaffective disorder, showed a statistically significant association between a low SOC and a high level of negative symptoms [14]. Another study of 120 patients with schizophrenia and schizoaffective disorder, showed a statistically significant association between low SOC and high levels of positive, negative and affective symptoms including anxiety, guilt feelings, tension and depressive mood [15].

Contemporary phenomenology distinguishes between the *core- or basic self and the narrative self* [16, 17]. The *basic self* is a pre-reflective level of selfhood. The basic self refers to the first-person quality of a person's experiences and is entirely implicit in–and inseparable from–the act of experiencing in itself, i.e. all subjective experience implies a self who is having the experience [18]. The concept of a basic self derives from the given fact that there is an implicit "ownership" of experience and in other words comprises the tacit awareness that "this is my experience". As the term indicate, the basic self is the foundation upon which other aspects of the self, such as the narrative self, are based. The narrative self exists on a reflective and articulated level. The narrative self is the experience of the self as having particular characteristics, personality traits, values and history [19].

Basic self-disturbances are disturbances in the basic, pre-reflective self [19]. BSDs affect a person's ability to have an adequate interpretation of his/her inner feelings and thoughts and diminish the feeling of ownership of one's body, movements and personal history. BSDs will thus likely affect how a person experiences his/her existence as comprehensive, manageable and meaningful, i.e. influence the person's SOC.

Both SOC and BSDs are closely linked to the clinical severity of mental illness [20–28], more knowledge about the relationship between SOC and BSDs give us insight into the challenges experienced by people with schizophrenia and other psychotic disorders. We here aim to investigate the relationship between BSDs and SOC in patients with psychotic disorders. To our knowledge, this is the first study that focuses directly this potential relationship. Our main hypothesis, as outlined above, is that patients with high level of BSDs will experience low SOC. Since high levels of BSDs are associated with high levels of symptoms and high levels of symptoms potentially associated with low SOC, we also investigate to what extent a putative association between BSDs and SOC is mediated by clinical severity, including the severity of symptoms of psychosis and functional loss.

## Material and method

### Sample

The current study is based on a seven-year follow-up of a cohort of first-treatment patients recruited in the period 2008–2009, in a sub-study of the Norwegian “Thematically Organized Psychosis” (TOP) study. The original inclusion criteria were: 1) Entering the first treatment for a broadly defined psychotic disorder a) schizophrenia, schizophreniform disorder or schizoaffective disorder (“SZspect") or b) psychotic bipolar disorder PBD I, II and NOS or delusional disorder and psychosis NOS (“‘non-SZspect”‘). 2) Age between 18 and 65 years and 3) IQ>70. Ninety patients met the criteria at baseline, for details see Haug et al. [29]. All participants were able and willing to consent. Ability to consent was determined in the following way: The patients were referred to the study by their treating clinician, who made the first evaluation of their ability to participate. The consent form was presented in both written and oral form to the patient by the interviewer, and questions regarding the study was discussed to make sure that the participant understood its content. Finally, the results of the symptom interviews, in particular the levels of psychotic symptoms, insight and disorganization were taken into account. Of the initial 90 patients recruited at baseline, 56 (62%) provided informed consent to participate in the follow-up study. All 34 patients who did not participate were alive; 19 did not want to participate, and we were unable to contact another 15 despite several attempts. The study was approved by the Regional Committee for Medical Research Ethics, South-East Norway.

### Assessments

All participants were evaluated with an extensive clinical assessment. Only the instruments relevant to this part of the study are presented below.

#### Assessment of Sense of Coherence (SOC)

SOC was measured with the SOC-13, a self-report questionnaire comprising thirteen questions. Each question links to one of the core components of the SOC (i.e. comprehensibility, manageability, and meaningfulness) [4]. Studies of the psychometric properties of the SOC-13 scale have shown high levels of validity and reliability [30], and internal consistency is found to be very good with Cronbach’s alphas ranging from 0.70 to 0.92 in the 127 studies using SOC-13 [2, 31].

In the current study, we used a version of SOC-13 modified to make it easier to understand for patients with severe psychiatric problems and previously used in a study of patients in acute psychiatric units in Norway [32]. The modified questionnaire asks the same questions as the original version but includes predefined answers on 1–5 scale instead of the original 1–7 Likert scale. The possible range of the SOC-13 score is thus 13 to 65, with high scores indicating good SOC.

#### Assessment of basic self disturbances

BSDs were assessed with the *Examination* of Anomalous Self Experience (EASE) manual [30] by the first author (IHS). IHS was trained in the use of EASE by EH (a certified EASE instructor) and PM (one of the original EASE authors). The inter-rater reliability (IRR) for the EASE assessments in the study was good [33–35].

The EASE manual is usually employed to capture the lifetime experience of BSDs, but the time range can be adjusted to the aim of the study [33]. Since one of the original study aims was to assess changes in BSD over time, we here measured BSDs experienced over the last two years before follow-up.

The EASE has 57 main items divided into five domains: (1) Cognition and stream of consciousness, (2) Self-awareness and presence, (3) Bodily experiences, (4) Demarcation/transitivism, and (5) Existential reorientation. BSDs is not conceptualized as discrete symptoms but as interconnected and highly overlapping aspects of a coherent Gestalt. Both items and domains are statistically highly inter-correlated because of overlap between single items and domains [23,28]. The items are scored using a 5-point scale (0–4), 0 = absent; 1 = questionably present; 2 = definitely present, mild; 3 = definitely present, moderate; 4 = definitively present, severe. The items were dichotomized into 0 (for absent or questionably present) and 1 (for definitely present comprising all severity levels) for the current analyses. Item 2.13 (anxiety) was not included in the analyses since this item is not a BSD per se [36] but primarily served as a contrast to the subsequent EASE-item (2.14), ontological anxiety [33].

#### Diagnostics and symptom evaluation

Diagnoses were ascertained by trained clinical psychologists or medical doctors using the Structured Clinical Interview for DSM-IV Axis I disorders (SCID module I, chapter A-E) [36]. An independent samples t-test showed no differences between the bipolar and other psychosis for the SOC score, the EASE total score or any EASE domain scores. These two participant groups were merged into one group, representing psychotic disorders outside of the schizophrenia spectrum (non-SZspect) for the statistical analyses.

Current symptom severity was measured with the Global Assessment Functioning Scale, Symptoms (GAF-S) [37, 38] and the Positive and Negative Syndrome in Schizophrenia Scale [39]. We used the Structured Clinical Interview for PANSS (SCI-PANSS) and report Wallwork’s five-factor model constituting positive, negative, disorganized, excitement and depressive symptoms [40]. PANSS item G12 (Lack of judgment and insight) was used as a measure of lack of clinical insight. Social functioning was assessed by the Global Assessment Functioning scale, Function (GAF-F) [37, 38] and the Social Function Scale (SFS). The SFS covers the area of social contacts, independent living and vocational activities, with a high level of internal consistency for the scales and subscales [41–43]. The first author (IHS) conducted all assessments except the diagnostic interviews.

#### Statistical analyses

All analyses were done using SPSS version 23.0 (SPSS Inc., Chicago, IL, USA. In addition to investigate the primary research question of an association between EASE and SOC, we did follow up analyses exploring associations between their subdomains (five in EASE and three in SOC). The p value was thus preset to p< 0.01 to adjust for multiple testing. Means and standard deviations are reported for continuous variables and percentages for categorical variables. We used bivariate correlations (Pearson’s r) to evaluate associations. Finally, a multiple linear regression analysis was used to assess the influence of clinical symptoms on the association between SOC and BSDs. The effect of potential confounders we evaluated through their bivariate associations with SOC and EASE). Based on a total N = 56 we restricted the number of independent variables to 5–6. Due to the high degree of association between different measures of symptoms, and between different measures of functioning, we chose to represent the two areas by the GAF-S and GAF-F scores, respectively. Substituting these with PANSS or SFS scores did not change the main results. Violations of assumptions of normality, homoscedasticity, linearity and multicollinearity were investigated for the final model.

## Results

Clinical and demographic data are shown in Table 1. The correlation matrix indicates a statistically significant correlation (*p<*0.01) between SOC and all clinical measures except PANSS NEG and PANSS DIS (Table 2). There were statistically significant differences (p < 0.01) in the EASE total score, GAF S, GAF F, PANSS POS and PANSS DIS between the two diagnostic groups, but no differences in the SOC-13 total score in bivariate analyses (Table 3).

**Table 1:** Clinical and demographic data.

	N	
Male (N/%)	56	28/50
Age (mean/median/SD)	56	32.2/29/7.4
Sense of Coherence (SOC) total score (mean/SD)	55	41.2/10.6
Examination of Anomalous Self-Experiences * (EASE) total score (mean/SD)	56	11.7/8.9
Global Assessment Functioning Scale split version–Symptom (GAF-S) (mean/SD)	56	57.2/16.8
Global Assessment Functioning Scale split version–Function (GAF-F) (mean/SD)	56	60.4/16.9
Positive and Negative Syndrome Scale (PANSS) total score (mean/SD)	56	50.7/13.5
Social Functioning Scale (SFS) (mean/SD)	53	107.6/10.4

**Table 2:** Correlation between SOC-13, EASE, clinical measures.

	EASE total score	GAF symptom	GAF function	PANSS POS	PANSS NEG	PANSS DEP	PANSS EXC	PANSS DIS	SFS total
SOC 13 total score	-0.544* p<0.001 N55	-0.516* p<0.001 N55	-0.407 p = 0.002 N55	-0.362 P = 0.007 N55	-0.175 p = 0.201 N55	-0.632* p<0.001 N55	-0.393 p = 0.003 N55	-0.037 p = 0.790 N55	0.483 p<0.001 N53
EASE total score		-0.671* p<0.001 N56	-0.425 p = 0.001 N56	0.633* p<0.001 N56**	0.227 p = 0.092 N56	0.662* p<0.001 N56	0.481* p<0.001 N56	.453* p<0.001 N56	-0.468* p<0.001 N54
GAF symptom			-0.791* p<0.001 N56	-0.694* p<0.001 N56	-0.545* p<0.001 N56	-0.644* p<0.001 N56**	-0.419 p = 0.001 N56	-.567* p<0.001 N56	-0.708* p<0.001 N54
GAF function				-0.491* p<0.001 N56	-0.622* p<0.001 N56	-0.425 p = 0.001 N56	-0.370 p = 0.005 N56	-.459* p<0.001 N56	-0.734* p<0.001 N54
PANSS POS					0.245 p = 0.069 N56	0.486* p<0.001 N56	0.491* p<0.001 N56	.529* p<0.001 N56	-0.447 p = 0.001 N54
PANSS NEG						0.229 p = 0.090 N56	0.09 p = 510 N56	.449 p = 0.001 N56	-0.695* p<0.001 N54
PANSS DEP							0.422 p = 0.001 N56	0.159 p = 0.243 N56	-0.490* p<0.001 N54
PANSS EXC								0.290 p = 0.030 N56	-0.228 p = 0.097 N54
PANSS DIS									-0.369 p = 0.006 N54

* Correlation is significant at the < 0.001 level (2-tailed).

SOC, Sense of coherence; EASE, Examination of Anomalous Self-experience; GAF, *Global Assessment Functioning Scale*; PANSS, Positive and Negative Symptom Scale; POS, Positive; NEG, Negative; DEP, Depressive; EXC, Excitative; DIS, Dissociative; SFS, Social Functioning Scale

**Table 3:** Independent samples t-test between the diagnostic groups (SZspect and non-SZspect).

		N	Mean	SD	t	*p-value*
SOC total score	Szspect	34	40,9	10,47	-0,35	0,725
	Non-SZspect	21	41,9	11,08		
EASE total score	Szspect	35	14,7	9,14	3,57	0.001
	Non-SZspect	21	6,8	5,78		
GAF symptom	Szspect	35	51,2	15,35	-3,84	**<0.001**
	Non-SZspect	21	67,1	14,54		
GAF function	Szspect	35	55,1	15,94	-3,31	0.002
	Non-SZspect	21	69,2	14,82		
SFS total score	Szspect	33	105,6	10,65	-2,44	0,018
	Non-SZspect	21	112,4	8,90		
PANSS POS	Szspect	35	8,1	3,65	4,62	**<0.001**
	Non-SZspect	21	4,9	1,62		
PANSS NEG	Szspect	35	12,4	4,72	2,47	0,017
	Non-SZspect	21	9,4	3,68		
PANSS DEP	Szspect	35	7,9	2,80	1,62	0,110
	Non-SZspect	21	6,5	3,25		
PANSS EXC	Szspect	35	4,9	1,25	1,59	0,117
	Non-SZspect	21	4,4	0,81		
PANSS DIS	Szspect	35	5,4	2,37	4,93	**<0.001**
	Non-SZspect	21	3,3	0,58		

There was a statistically significant (negative) correlation between the SOC-13 total score and the EASE total score (r-0.54/p <0.001) (Table 4). The follow-up analyses indicated statistically significant negative correlations between the main parts of the EASE domain scores and the SOC components, with the highest r's for the associations between SOC and EASE domain 2, Self-awareness and presence (Table 4, in bold).

**Table 4:** Correlations (Pearson’s r) between SOC-13 and EASE scores.

	EASE Total Score r/p-value	Domain 1 r/p-value	Domain 2 r/p-value	Domain 3 r/p-value	Domain 4 r/p-value	Domain 5 r/p-value
SOC components						
Comprehensibility	-0.51^/^<0.001	-0.40/0.003	**-0.55/<0.001**	-0.42/0.002	-0.30/0.025	-0.32/0.019
Manageability	-0.45/0.001	-0.36/0.004	**-0.41/0.002**	-0.32/0.018	-0.40/0.003	-0.26/0.06
Meaningfulness	-0.46/<0.001	-0.28/0.04	**-0.54/<0.001**	-0.35/0.009	-0.19/0.16	-0.35/0.009
SOC-13 Total Score	-0.54/<0.001	-0.41/0.002	**-0.55/0.001**	-0.42/0.002	-0.33/0.014	-0.36/0.007

We then investigated if clinical characteristics mediated the association between SOC and BSDs (EASE total score) (Table 5).

**Table 5:** Multiple linear regression models with SOC-13 total score as dependent variable.

	Beta	t	p-value	95.0% CI		Collinearity Statistics	
				Lower	Upper	Tolerance	VIF
Model 1							
(Constant)		24.3	< 0.001	44.76	52.803		
EASE total score	-0.544	-4.72	< 0.001	-0.93	-0.375	1.000	1.000
Model 2							
(Constant)		5.70	< 0.001	34.54	72.12		
GAF-F	0.177	0.82	0.42	-0.16	0.39	0.24	4.19
GAF-S	0.079	0.43	0.67	-0.19	0.29	0.32	3.11
Insight	-0.223	-1.75	0.09	-6.73	0.46	0.69	1.46
Diagnoses	-0.344	-2.81	0.006	-12.79	-2.11	0.74	1.35
EASE total score	-0.498	-3.32	0.002	-0.96	-0.24	0.50	2.01

CI indicates Confidence Interval; EASE, Examination of Anomalous Self-experience; GAF- F Global Assessment Functioning scale, Symptom; GAF-S, Global Assessment Functioning scale, Function; Insight, PANSS item G12 (Lack of judgement and insight); Diagnoses, SZspect vs. Non-SZspect

The results of the regression analysis showed a statistically significant association between SOC and BSDs, in the direction that high levels of BSDs contributed to low levels of SOC. The analysis also indicated that the association between SOC and BSDs was independent of diagnostic group, and not mediated by levels of clinical symptoms or dysfunction. The level of clinical insight did not confound the analyses.

## Discussion

In line with our hypothesis, we found a statically significant association between SOC and BSDs, in the direction that patients with high levels of BSDs had low levels of SOC. The association was strongest for EASE domain 2: Self-awareness and presence. As expected, we also found associations between SOC, BSDs and clinical characteristics. However, the association between SOC and BSDs was not mediated by clinical symptoms, dysfunction, level of insight or diagnostic group.

BSDs comprises a disordered experience of thoughts, feelings, and sensations with consequential fundamental loneliness, despite access to relationships with others [26, 44, 45]. This counteracts trust in- and access to the person's own and others' resources and abilities, that are needed to handle difficult situations. The experience of loss of common sense makes everyday life appear as complicated and unnatural. Even trivial tasks have to be thought through in a deliberate and careful (rather than an automatic and intuitive) fashion [19, 46, 47], creating uncertainty around the performance of actions others take for granted. The experience that life is coherent, consistent and predictable is thus influenced in a negative way. BSDs also comprise a diminished first-person perspective, disrupting awareness of own actions and reducing the ability to be touched, moved or motivated by other people, events or situations [48]. This impaired awareness may weaken the experience of personal involvement in own life and the experience of life as meaningful.

In particular EASE domain 2 (i.e. self-awareness and presence) comprises changes in the experience of a normally tacit and pre-reflective self-presence. The items included in this domain are thus associated with diminished potentials for activity and pleasure, diminished engagement in the world and experiences of alienation [33]. The SOC concept includes predictability, manageability and meaningfulness. It is difficult to experience life as predictable in the context of experiencing the world as unnatural and strange, to experience life as manageable in the context of actively monitoring inner life and external reality and to experience meaningfulness in the context of severe estrangement.

Significant associations between two apparently different concepts can be based on overlapping criteria. There are, however, major differences in the way BSDs and SOC are assessed. The EASE manual is a comprehensive, in-depth interview made to elicit information about BSDs as complex, and often unexpressed, experiences or phenomena. The interviewer explores these phenomena together with the patient beyond the immediate answer to the question. The use of a particular word or metaphor is examined to grasp the patient’s interpretation of the underlying phenomenon that can be literal, concrete or outside of the usual understanding of a concept [33]. The SOC-13, on the other hand, is a short self-report questionnaire assessing the person's global experience of comprehensibility, manageability and meaningfulness [4]. The self-report form precludes any further exploration of the answers, and mainly captures the person’s immediate response to the question. There are questions in the SOC-13 and EASE-interview that appear closely related at first glance. SOC: "How often do you have the feeling that you are in an unfamiliar situation and do not know what to do?” EASE: “Have you ever experienced that you no longer understand natural things in life, that it is difficult to understand situations, people and objects?” The follow-up clarifications in the EASE interview, however, counteract the use of overlapping criteria.

The findings also support the notion that BSDs and SOCs relate to different types of self-awareness: While BSDs are associated with the basic level of the self, SOC is associated with the narrative level. This assumption is the basis for interpreting BSDs as independent contributors to poor SOC, in patients with psychosis. The results of the multiple linear regression analyses also indicate that the association between BSDs and SOC is not confounded or mediated by clinical symptoms or social dysfunction. However, the correlational nature of this study precludes firm conclusions regarding the direction of the associations, or causality. SOC theory is used to explain the apparent contradiction that people with severe illnesses have a subjective experience of good health. In this study, there was a negative association between the level of symptomatology and the level of SOC. It is also of interest that lack of clinical insight contributed to a lower SOC, and did not influence the association between BSDs and SOC. Also, while some participants reported both high symptom levels and high SOC, this discrepancy was not explained by a lack of insight.

Experience of health is linked to personal history and influenced by access to resources and the impact of illness. Both BSDs and SOC captures subjective experiences and needs to be specifically explored by clinicians wanting to understand the subjective world of their patients. By taking a salutogenic position, the clinician and the patient can explore how different challenges in life, including the presence of BSDs, impact the experience of health and coherence. This exploration can serve as the basis for identifying resources and initiating interventions that strengthen the patient's experience of health.

Strengths of this study include: That the sample was broadly recruited from the Norwegian national health service offering public mental health care to all individuals with mental illness with few exclusion criteria. The participants represent a comprehensive, near to epidemiological sample.

Limitations include: While the current SOC-13 form is thought to be easier to use for patients with mental disorders, the use of the original form with a seven-point Likert scale could potentially give richer data through a wider range of scores. In addition, the study has no explicit measure of the subjective experience of health, which could have strengthened the study.

## Conclusion

This study is the first to demonstrate a statistically significant association between BSDs and SOC. The association is particularly high between SOC and the EASE domain 2, self-awareness and presence. The association is not influenced by diagnostic category, clinical symptoms and social dysfunction. Further research exploring this relationship, including the direction of the association, potential indications of causality and implications for treatment is needed.
